# Clinical presentations of hypertrophic cardiomyopathy and implications for therapy

**DOI:** 10.21542/gcsp.2018.19

**Published:** 2018-08-12

**Authors:** Carles Díez-López, Joel Salazar-Mendiguchía

**Affiliations:** 1 Advanced Heart Failure and Heart Transplant Unit. Heart Institute. Bellvitge University Hospital. Barcelona, Spain; 2 Health in Code. Cardiovascular Genetics Department. A Coruña, Spain; 3 Genetics Department. Universitat Autònoma de Barcelona, Spain

## Introduction

Hypertrophic cardiomyopathy (HCM) is diagnosed in the presence of left ventricular hypertrophy of ≥15 mm in adult index cases, or ≥13 mm in relatives of known affected patients, that is not solely explained by abnormal loading conditions^1, 2^. In children, the left ventricle (LV) wall thickness should be more than two standard deviations above the predicted population mean^1^. The typical anatomo-pathological findings include myocyte hypertrophy, disarray, interstitial fibrosis and small-vessel disease^3^. Although all the myocytes are supposed to be affected, pathological alterations are not uniformly distributed throughout the myocardium^4^. Asymmetrical hypertrophy of the interventricular septum is the most commonly observed phenotype, but any pattern of hypertrophy is consistent with the diagnosis^5^.

## Aetiology

HCM is mainly caused by mutations in genes encoding for sarcomeric proteins, which account for almost 60% of the cases^6^. It has been estimated that up to 10% of patients with HCM present with other genetic or non-genetic phenocopies^7, 8^, but up to 30% of HCM cases remain unexplained^6^.

Common diagnostic dilemmas include the differentiation of HCM from physiological hypertrophy in athletes^9–11^ and conditions such as systemic hypertension or valvular heart disease. People of sub-Saharan African ancestry may be more prone to developing myocardial hypertrophy, even in the absence of severe predisposing factors^12^. It is important to correctly distinguish between these phenocopies and HCM to prevent fatal outcomes, not only for the index cases, but also for affected family members

Rare, but important, causes of HCM include infiltrative disorders like amyloidosis, lysosome and glycogen storage diseases, and mitochondrial diseases^13–17^. In children, neuromuscular disorders, inborn errors of metabolism, RASopathies or malformation syndromes are more prevalent^18, 19^. Many of these disorders display disease-specific clinical features that, together with the age of presentation, can assist in the diagnostic work-up^13^. Early identification of these conditions facilitates a tailored approach to disease management, which in some cases, may lead to significant modifications of prognosis and quality of life^1, 13^.

## Clinical manifestations and therapeutic implications

Clinical manifestations in HCM are highly variable^20^. Classical symptoms in HCM are usually related to left ventricular outflow tract (LVOT) obstruction, mitral regurgitation, myocardial ischemia, diastolic dysfunction, abnormal vascular responses, and supraventricular and ventricular arrhythmias^20, 21^.

### Dyspnoea

Reduced functional capacity is common in HCM. Orthopnea and paroxysmal nocturnal dyspnoea are rare, but bendopnoea and postprandial breathlessness are common symptoms. LVOT obstruction is a common cause of these clinical manifestations, but exertional dyspnoea is also caused by diastolic and systolic LV impairment and atrial arrhythmia^20^.

The first approach to LVOT obstruction is based on medical treatment optimization^1, 2^. It is important to ensure adequate ventricular filling and relaxation and to avoid vasodilators and inotropic agents^20, 22^. Selective beta-blocking agents are considered the gold standard therapy. Verapamil and diltiazem are considered less effective, although they can be used in patients who are intolerant or have contraindications to beta-blockers^20, 22^. It is important to monitor ECG changes when using disopyramide to avoid QT prolongation, and although generally tolerated, patients should be warned of cholinergic side-effects. Verapamil or diltiazem are also considered options for the treatment of LV obstruction, and can be used in conjunction with beta-blockers under close monitoring^20, 22^. For drug-refractory patients, septal reduction (with surgery or alcohol septal ablation) may be considered^20^. In experienced centers, septal reduction shows marked symptom improvement without significant intraoperative morbidity and mortality^23–26^. The use of atrio-ventricular sequential pacing can also be considered but is generally less effective^27, 28^.

Mavacamten^©^ (MYK-461, Myokardia Inc., San Francisco, California, United States) is a small molecule that reduces the steady-state ATPase activity by inhibiting the rate of phosphate release of *β*-cardiac myosin^52^. This results in diminished hypercontractility in the sarcomere and a consistent reduction in LV obstruction and symptoms, which is expected to persist in mid-term follow-up^53^. The phase 2 PIONEER-HCM (A Phase 2 study of Mavacamten (formerly MYK-461) in Symptomatic Obstructive Hypertrophic Cardiomyopathy Patients) clinical trial has completed without significant safety concerns. The PIONEER-HCM, the EXPLORER-HCM and the MAVERIK-HF trials, will evaluate the effect in different phenotypes and larger cohorts.

Many patients can have severe symptoms in the absence of significant LV outflow tract obstruction^29^. Exercise echocardiography should be considered in such patients to rule out provocable LVOT obstruction, mitral regurgitation or diastolic impairment with pulmonary hypertension^20^. The principle mechanism of symptoms in non-obstructive patients is diastolic dysfunction and much less commonly mitral regurgitation caused by intrinsic mitral valve abnormalities. It is important to perform a comprehensive study to determine the exact mechanisms of symptoms and define the best treatment strategy. Left atrial enlargement and pulmonary hypertension are usually related to the degree of diastolic impairment in HCM patients^29^. In the absence of concomitant obstructive haemodynamics, the addition of diuretics can help in some situations^20^. To date, several trials have tried to modify the disease course via blockade of the renin-angiotensin-aldosterone axis with little effect.

### Chest pain

Angina in HCM is most commonly caused by obstruction and microvascular coronary abnormalities^30^. Nonetheless, it is important to rule out significant epicardial coronary artery disease, particularly in older populations and patients with cardiovascular risk factors^1, 2^ using coronary angiography (either by CT coronary angiography or cardiac catheterization)^20^. In addition, cardiac catheterization can provide relevant information on coronary artery bridging and the anatomy of the septal arteries that can be useful in refractory patients who are candidates for alcohol septal ablation. Verapamil and diltiazem are the preferred drugs to treat chest pain, in the absence of systolic dysfunction, as they are thought to improve microvascular blood flow^20^. Nitrates can be used with caution in the absence of an LVOTO^1^.

A number of drugs that alter myocardial energy metabolism have been evaluated in HCM. The RESTYLE-HCM (ranolazine in patients with symptomatic hypertrophic cardiomyopathy) trial, failed to demonstrate benefit in exercise performance or quality of life despite showing a reduction in ventricular premature complexes^31^. Perhexiline, a carnitine palmitoyltransferase (CPT)-1 inhibitor that improves myocardial performance by creating a switch from fatty acid to glucose metabolism^32^, has been shown to improve HCM symptoms by improving myocardial energetics, but safety issues regarding hepatitis and neuropathy has limited its widespread use^33^.

### Syncope and arrhythmias

Symptoms related to brief loss of consciousness may appear in one of every five HCM patients, but it is hard to identify the precise mechanisms in most of cases, in spite of a detailed comprehensive study^34^. Typical mechanisms include left ventricular outflow tract obstruction, abnormal vascular responses and arrhythmias^34^.

The incidence of atrial fibrillation (AF) is estimated to be around 3% in HCM, and is considered the most common sustained arrhythmia in HCM^35^. AF is most commonly paroxysmal and the life-long prevalence is estimated to be around 30%^36^. The incidence increases in the presence of other concomitant risk factors like hypertension, dyslipidemia, diabetes mellitus or sleep apnoea^35, 37^. The development of AF is related to atrial electrical and structural remodeling, which in turn relates to the severity of ventricular hypertrophy and left atrial size^38^. AF in HCM is often accompanied by clinical deterioration and is related to adverse cardiovascular events^35^. The hemodynamic deterioration is presumed to be due to rapid ventricular rate and loss of atrial contribution, causing inadequate filling and exacerbating LVOT obstruction. The implementation of early and aggressive rhythm control strategies using antiarrhythmic drugs and in selected cases radiofrequency AF ablation is essential^39^. Long-term anticoagulation is recommended for all patients with HCM patients who present with AF, irrespective of predictive risk scores such as CHA2DS2-VASc score^35, 40^. Vitamin-K antagonists are considered the first choice in this setting^35^, although direct anticoagulants are suitable alternatives for thromboembolic prevention, and might be preferred in selected individuals^41^.

Non-sustained ventricular tachycardia occurs in almost one third of patients during the follow-up and is considered a major risk factor for adverse events, particularly sudden cardiac death (SCD)^42, 43^. The intrinsic mechanisms involved in the development of arrhythmias include hypertrophy, ischemia, fibrosis, myocyte disarray and electric uncoupling^43, 44^. Certain mutations may be associated with SCD^43^, and consequently, genotyping may be considered as part of the risk stratification in some specific cases^43^.

Antiarrhythmic drugs have no role in the primary prevention of SCD but are useful in the rare patient with symptomatic ventricular tachycardia. Catheter ablation for VT is also feasible in drug-refractory patients, although the best results are achieved by using a combined endo and epicardial approach^44^. ICDs should be considered in all patients with symptomatic VT^20, 45^.

## Clinical phenotypes in hypertrophic cardiomyopathy

**Figure 1.**
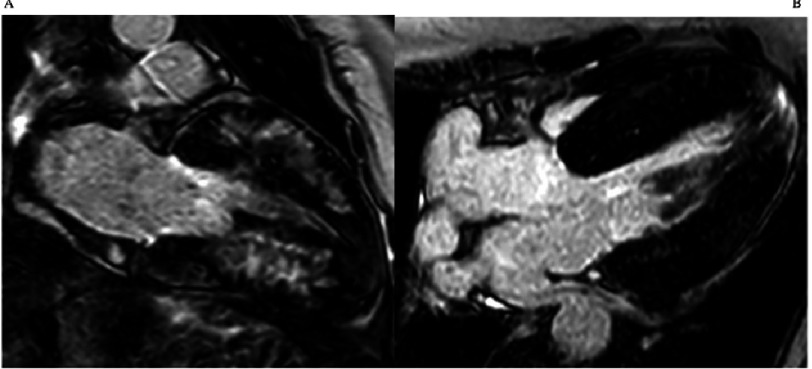
Cardiac Magnetic Resonance (CMR) late gadolinium enhancement images: Classical hypertrophic cardiomyopathy in an individual who suffered a sudden cardiac death. MYBPC3 c.2149-1G>A. A) 2 chamber view with severe hypertrophy in the segments of maximum hypertrophy. B) 4 chamber view showing biventricular hypertrophy, apical insertion of the papillary muscles and slight late gadolinium enhancement in the apical segment.

The classical HCM phenotype includes asymmetric hypertrophy, that predominantly affects the interventricular septum^46, 47^ (Figure 1). Typical ECG findings include LV hypertrophy, deep and wide Q waves, and negative T waves. A normal ECG is present in around 5% of patients. Pathogenic sarcomeric gene mutations are most common in patients with this classical phenotype^48–50^.

**Figure 2.**
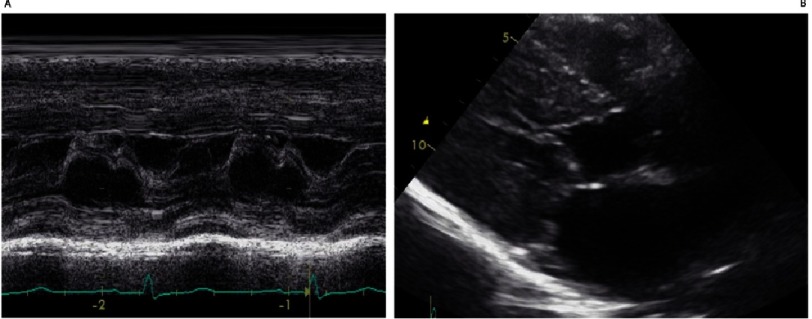
A) M-Mode echocardiography: Classical systolic anterior movement (SAM) of the mitral valve causing obstruction. B) B-Mode Echocardiography: Paraesternal longitudinal axis showing contact between interventricular septum and the anterior leaflet of the mitral valve in the beginning of systole.

Patients with the classical phenotype are more predisposed to have dynamic subaortic obstruction caused by systolic anterior movement (SAM) of the mitral valve (Figure 2)^20^. The mitral valve the subvalvular apparatus may be abnormal and contributes the development of LVOT obstruction and mitral regurgitation^51^. In childhood, LVOT obstruction is common, and in some cohorts it has been reported to occur in around 50% of the patients. Interestingly, it occurs in the presence of greater degrees of hypertrophy and smaller left ventricular volumes than in the adult cohorts^18^. LVOTO is associated with morbidity and mortality in terms of disease progression, heart failure symptoms and cardiovascular events^20^.

### Non-classic HCM phenotypes

Hypertrophy may involve any location of the ventricular wall^48^. Although some of these less typical phenotypes have been related to mutations in the thin-filaments, casual mutations may involve any the sarcomere genes^6^.

**Figure 3.**
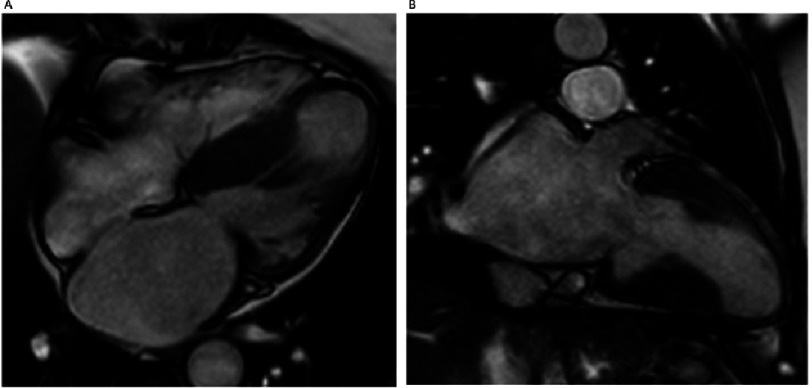
Cardiac Magnetic Resonance (CMR) cine apical 4 and 2 chamber view showing apical aneurism and mid-cavity hypertrophy. TNNI 3 p.Ala157Val.

Mid-ventricular hypertrophy in conjunction with abnormal distribution of the papillary muscles and hypercontractility of the lateral ventricular wall generating a mid-cavity obstructive gradient occurs in less than 10 % of cases^54^, but is particularly important as a cause of symptoms^55^. Mid-cavity obstruction may correlate with end-stage disease progression and development of an apical aneurysm. Stroke from thrombus formation and ventricular arrhythmias can occur in these patients, and the phenotype may be associated with cardiovascular death^20^. (Figure 3)

**Figure 4.**
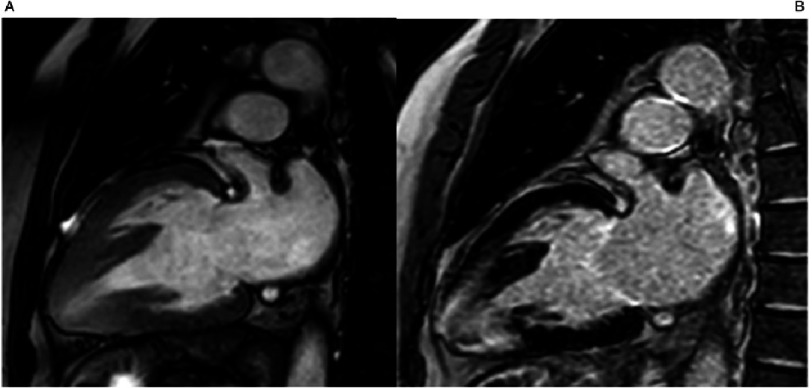
Cardiac Magnetic Resonance (CMR) Apical 2CH view: A) Cine: Apical hypertrophic cardiomyopathy with the classical “ace of spades” shape. B) Late gadolinium enhancement images: severe fibrosis in the segments of maximum hypertrophy.

The apical hypertrophic phenotype is associated with deep negative T waves in precordial leads, and the “ace of spades” appearance on ventriculography^56^. It is often overlooked because of poor apical visualization on standard echocardiographt and may require echo contrast agents or CMR for identification^48^. Patients with apical hypertrophy have been considered to have a benevolent course, but the evidence is contradictory^57^. Indeed diastolic impairment, left atrial enlargement and impaired excercise capacity, might be frequent on apical forms^58, 59^. (Figure 4)

### Genotype positive and phenotype negative HCM

Next generation sequencing and family screening have resulted in the identification of a large number of relatives with mild or subclinical disease. Electrocardiographic markers and minor echocardiographic changes may be helpful in diagnosing such individuals when they are relatives of clearly affected HCM patients^60^. Minor abnormalities seen in relatives include low end-systolic volumes, mild contractile and diastolic alterations, increased myocardial trabeculation, limited late gadolinium enhancement and an increase in the extracellular volume fraction^20^.

In a recent study, 30% of evaluated relatives were identified as having HCM at first screening. Moreover, 16% developed HCM during 7 years of repeated evaluation. Therefore, genotype positive patients need prolonged surveillance although adverse events were extremely infrequent in relatives without a clinically evident phenotype in the presence of a positive genotype^61^. Current recommendations are to perform screening at yearly intervals in children and adolescents until their mid twenties and at 3–5 year intervals in older individuals^20^.

### End-stage HCM

**Figure 5.**
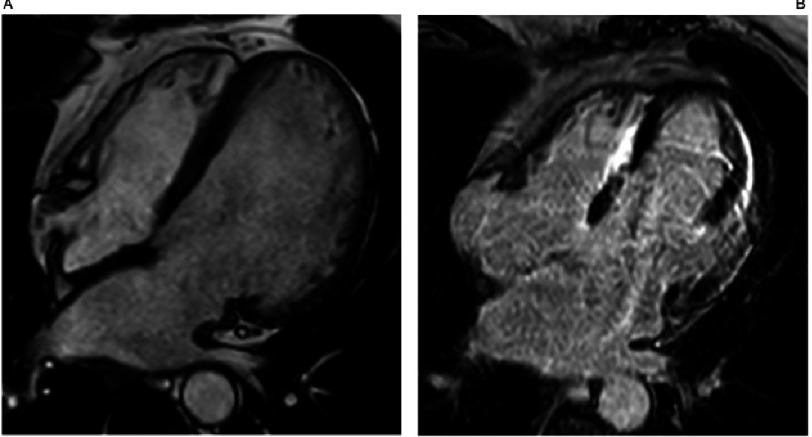
Cardiac Magnetic Resonance (CMR) Apical 4CH view: A) Cine and B) Late gadolinium enhancement images. End-stage HCM with progressive remodelling and sever systolic dysfunction, atrial fibrillation and ventricular tachycardia. MYH7 p.Arg453Cys.

**Figure 6.**
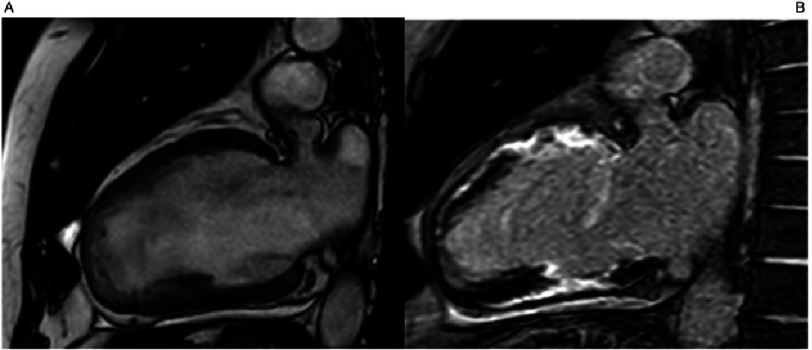
Cardiac Magnetic Resonance (CMR) Apical 2CH view: A) Cine and B) Late gadolinium enhancement images. End-stage HCM with progressive remodelling and sever systolic dysfunction, atrial fibrillation and ventricular tachycardia. MYH7 p.Arg453Cys.

**Figure 7.**
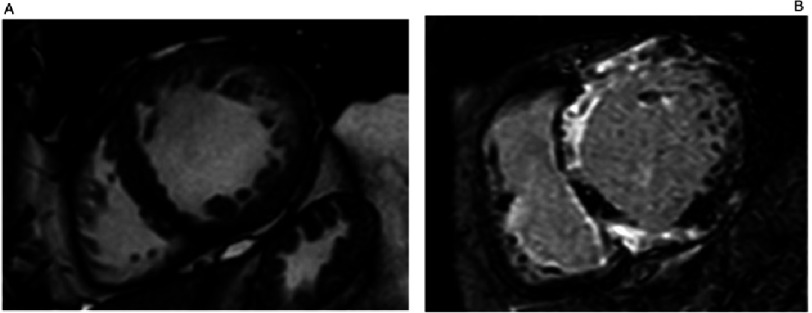
Cardiac Magnetic Resonance (CMR) Apical short axis view: A) Cine and B) Late gadolinium enhancement images. End-stage HCM with progressive remodelling and severe systolic dysfunction, atrial fibrillation and ventricular tachycardia. MYH7 p.Arg453Cys.

Progressive systolic dysfunction, often accompanied by ventricular dilatation and left ventricular wall thinning occurs in 2.5% to 15% of patients^62^. End-stage HCM with LV dilation and systolic dysfunction is the most common clinical profile of patients undergoing heart transplantation^63^. In these advanced stages of the disease, patients usually present with mixed phenotypes displaying different degrees of remodelling, restrictive filling patterns and systolic dysfunction^20^. Furthermore, left ventricular trabeculae and non-compaction is not rare, further complicating the clinical diagnosis (Figures 5 and 6). Rarely, patients may be indistinguishable from dilated cardiomyopathy in advanced cases (Figure 7). Severe diffuse fibrosis is one of the hallmarks of end-stage disease^62^. Many factors have been proposed to account for end-stage disease including gene mutation, environmental and epigenetic factors^20^.

Treatment of HCM patients who develop LV systolic dysfunction should be according to the standard heart failure guidelines with a few provisos^64^. Patients should be referred early for ICD implantation for SCD prevention because the rate of ventricular arrhythmias has been shown to be consistently higher in this population^62^. Cardiac resynchronization therapy has not consistently shown to improve left ventricular systolic function or survival in these patients^65^. Long-term mechanical circulatory support in selected cases with refractory heart failure as a bridge to transplant strategy is feasible^1^. In this regard, the use of continuous flow devices might be restricted to severely dilated ventricles to allow satisfactory support and avoid device complications.

Finally, patients with cardiomyopathies tend to be younger than common HF patients, and are able to tolerate advanced stages of the disease with apparent good exercise performance. Accordingly, it is important to routinely refer these patients to experienced centres, in order to perform a comprehensive heart failure risk stratification.

## Conclusions

In the last 50 years, the knowledge of HCM pathophysiological features and its clinical perspective has increased enormously. It is possible to make an aetiological diagnosis in more than two-thirds of patients, with implications for clinical management, familial screening and preclinical investigation. Early disease detection facilitates early implementation of treatment strategies to prevent disease complications.
